# Complete genome sequence of *Pseudomonas* sp. PP3, a dehalogenase-producing bacterium, confirms the unusual mobile genetic element *DEH*

**DOI:** 10.1128/mra.00089-25

**Published:** 2025-04-21

**Authors:** Gordon Webster, Amy J. Baldwin, Edward Cunningham-Oakes, Alex J. Mullins, Rachel Dodds, Katja E. Hill, Li Ling Lee, Mark J. Leggett, Andrew W. Topping, Andrew J. Weightman

**Affiliations:** 1 Microbiomes, Microbes and Informatics Group, Organisms and Environment Division, School of Biosciences, Cardiff University 151285, Cardiff, Wales, United Kingdom; 2 Department of Chemistry, University of Warwick 201055, Coventry, England, United Kingdom; The University of Arizona, Tucson, Arizona, USA

**Keywords:** dehalogenase, biodegradation, mobile genetic elements, *Pseudomonas*, bioremediation

## Abstract

*Pseudomonas* sp. PP3, originally isolated from contaminated soil enriched in a chemostat culture on 2,2-dichloropropionic acid, has a 6.42 Mb genome, most closely related to *P. reinekei*. This well-characterized organism continues to provide key insights into adaptive dehalogenase-mediated bioremediation of halogenated organic pollutants.

## ANNOUNCEMENT

Halo-organic compounds are used as herbicides, pesticides, preservatives, solvents, and other applications. The persistence and toxicity of many of these compounds raise serious environmental concerns regarding their use (1). Biodegradation of such compounds, including haloalkanoic acids, by *Pseudomonas* species is well known (2).

*Pseudomonas* sp. strain PP3 was isolated from a soil microbial community in a chemostat culture on the herbicide 2,2-dichloropropionic acid as the sole carbon and energy source as described (3–5). For long-term storage, the strain was kept in 40% (vol/vol) glycerol stocks at −80°C and for routine laboratory culture grown aerobically in standard basal salts medium [SBS; (4)] containing 5 mM halogenated substrate at 30°C, shaking at 150 rpm.

For genome sequencing, PP3 was grown in SBS with 10 mM 2-chloropropionic acid for 24 h. Cells were pelleted by centrifugation (4,000 rpm, 10 min, ALC-PK120 centrifuge), and genomic DNA was extracted using the Wizard genomic DNA purification kit (Promega) according to the manufacturer’s protocol. Following QC, DNA was sheared and size-selected (~10 kb) using Covaris g-TUBE and sequenced by Novogene (UK). Sequencing libraries were prepared with the SMRTbell template prep kit 1.0 (PacBio) and NEBNext DNA library prep kit for Illumina. Genome sequencing was performed on a PacBio Sequel SMRT Cell 1M and an Illumina NovaSeq 6000 (paired-end, 2 × 150 bp). In total, 208,470 high-quality PacBio subreads (average length = 5,218  bp; N50 = 7,983  bp) and 19,253,220 Illumina raw reads were obtained and quality checked with FastQC v0.11.8. The genome (620× coverage) was assembled *de novo* from the PacBio subreads into one contig using Flye v2.8 (6) and polished with both the PacBio reads using Arrow (via pbmm2 v1.4.0, GCpp v2.0.0 tools; https://github.com/PacificBiosciences), and Illumina reads with Pilon v1.23 (7) using default settings. The assembled genome was reoriented with Circlator v1.5.5 (8) at the *dnaA* gene start position.

**Fig 1:**
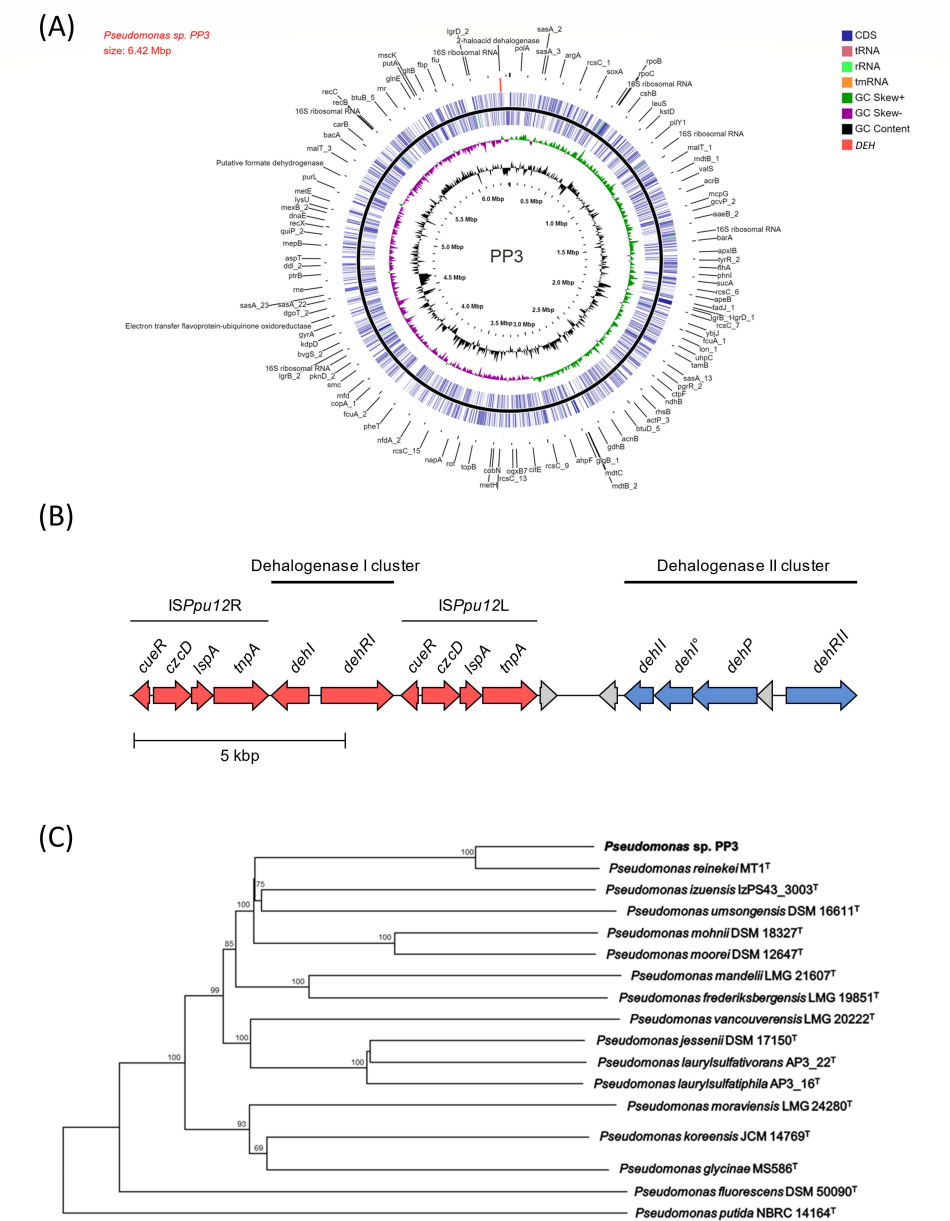
Visual representation and phylogenetic classification of the *Pseudomonas* sp. PP3 genome and *DEH* mobile element. (**A**) *Pseudomonas* sp. PP3 chromosome map of 5,744 predicted proteins. From outer circle to the center: select view of gene identification; dehalogenase gene clusters (within and around *DEH*); all CDSs (blue) and RNAs (crimson/green/orange) genes on forward strand; all CDSs (blue) and RNAs (crimson/green/orange) genes on reverse strand; GC skew (positive GC skew values are plotted in green, and negative values are in purple); GC content (black); scale bar. The map was generated using the Circular Genome Viewer (CGView). (**B**) Close-up of the dehalogenase gene clusters (*dehI*) and the upstream *dehII* region highlighted in (**A**), generated using Clinker v0.0.27 (14). The gene designations for the dehalogenase I cluster within the *DEH* element are as follows: *dehI*, dehalogenase I family gene (12); *dehRI*, σ^54^-dependent activator; *tnpA*, putative ISL3-family transposase; *lspA*, putative lipoprotein signal peptidase; *czcD*, putative heavy-metal-associated efflux transporter; *cueR*, putative heavy-metal-associated responsive transcriptional regulator. The positions of the IS*Ppu12* insertion sequence (independently mobile) flanking regions are also indicated (13). The gene designations for the dehalogenase II cluster are as follows: *dehII*, dehalogenase II family gene; *dehI°*, cryptic dehalogenase I family gene; *dehP*, putative permease transporter; *dehRII*, σ^54^-dependent activator. (**C**) Phylogenetic classification of *Pseudomonas* sp. PP3. The genomes of the 14 closest related type strains along with type strains *P. putida* and *P. fluorescens* were used for phylogenetic analysis as described by TYGS (15). The tree was inferred with FastME 2.1.6.1 (16) using Genome BLAST Distance Phylogeny (GBDP) distances calculated from genome sequences. The branch lengths are scaled in terms of GBDP distance formula *d*_5_. The numbers above branches are GBDP pseudo-bootstrap support values > 60% from 100 replications, with an average branch support of 86.1%. The tree was rooted at the midpoint.

Genome size and other metrics for the assembly are as follows: 6,421,237 bp and 59.17%G + C, similar to other *Pseudomonas* species (Table 1; 9), 5,745 coding DNA sequences (CDS), 19 rRNAs, 71 tRNAs, and four ncRNAs identified using PGAP v6.7 (10) and CGView (11) (Fig. 1A). The PP3 genome contains two 2-haloalkanoate dehalogenase genes (representing *dehI* and *dehII* gene families) previously described (12), and one silent *dehI* gene. The *DEH* mobile genetic element in PP3 (13) is confirmed to contain a *dehI* family gene (12) and its regulatory gene, *dehRI,* flanked by two almost identical insertion sequences (IS*Ppu12*). The *DEH* element is located close to a separate putative dehalogenase operon (Fig. 1B). Several other ORFs encoding putative dehalogenases and enzymes associated with halo-organic catabolism are also evident in the genome.

**TABLE 1:** Pairwise ANI and digital DDH between *Pseudomonas* sp. PP3 and other *Pseudomonas* species, with their respective genome sizes and %G + C content

Genome (accession number)	Genome size (Mbp)	G + C content (%)	*Pseudomonas* sp. PP3	
			Pairwise ANI (%)^*a*^	Pairwise DDH (%)^*b*^
*Pseudomonas* sp. PP3	6.42	59.2	100	100
*Pseudomonas reinekei* MT1^T^ (GCA_001945365)	6.25	59.2	95.6	63.3
*Pseudomonas izuensis* IzPS43_3003^T^ (GCA_009861505)	6.86	59.6	88.5	33.6
*Pseudomonas umsongensis* DSM 16611^T^ (GCA_002236105)	6.70	59.7	88.2	33.0
*Pseudomonas mohnii* DSM 18327^T^ (GCA_900105115)	6.59	59.6	88.3	33.5
*Pseudomonas moorei* DSM 12647^T^ (GCA_900102045)	6.55	59.7	88.5	34.0
*Pseudomonas putida* NBRC 14164^T^ (GCA_000412675)	6.16	62.3	84.3	22.4
*Pseudomonas fluorescens* DSM 50090^T^ (GCA_001269845)	6.39	60.2	85.6	24.7

^*a*^ Average nucleotide identity (ANI) values <95% indicate different species.

^*b*^ *In silico* DNA-DNA hybridization (DDH) values <70% indicate different species (17).

Average nucleotide identity (ANI) analysis with pyani v0.2.10 (18) and genome comparisons using the Type (Strain) Genome Server [TYGS; (15)] inferred that PP3 groups phylogenomically with *P. reinekei* MT1^T^ (Table 1; Fig. 1C), with an ANI value >95% proposed as the same species (17). However, dDDH analysis produced a value below the 70% species threshold (17) compared with *P. reinekei* and other *Pseudomonas* genomes (Table 1). The ANI and dDDH values together suggest that PP3 may represent a new species closely related to *P. reinekei*.

## Data Availability

The genome sequences and Illumina raw sequence reads were deposited via the European Nucleotide Archive (ENA) under the ENA project/study number PRJEB43414. The accession numbers for the PP3 genome assembly and raw reads are GCA_905336995 and ERX5224659, ERX5224704, ERX5225337 and ERX5225391, respectively.
